# Immunohistochemical Markers in Endometrial Cancer

**DOI:** 10.3390/cancers13030505

**Published:** 2021-01-29

**Authors:** Valerio Mais, Michele Peiretti

**Affiliations:** 1 Department of Surgical Sciences, University of Cagliari Medical School, 09042 Cagliari, Italy; 2 Division of Gynecology and Obstetrics, Department of Surgical Sciences, University of Cagliari, 09042 Cagliari, Italy; mpeiretti@aoucagliari.it

In 2018, 382,069 new cases of uterine cancer were registered worldwide and 89,929 deaths from this cancer were reported [1]. This cancer is the sixth most common cancer in the female population worldwide, with an incidence that is more than three-fold greater in regions of the world with a high or very high human development index (HDI) than in regions with a low or medium HDI [1]. Moreover, cancer of the uterine body is the thirteenth deadliest cancer in the female population worldwide, with a mortality rate that is twice as high in regions of the world with a high or very high HDI compared with that in regions with a low or medium HDI [1]. The prognosis of endometrial cancer depends on the stage, histology, grade and ethnicity of the woman [2]. In fact, endometrial cancer mortality appears to be much higher in Black women than in Caucasian, Hispanic and Asian women, also because the former are significantly more frequently affected by endometrial cancer with a histology other than low-grade endometrioid, which is most often diagnosed at an early stage [2].

For low-grade endometrioid endometrial cancer, the standard treatment is surgery, and when surgery confirms an early stage, surgical treatment is also the only treatment [3]. The staging of endometrial cancer has been surgical since 1988 and involves systematic pelvic and para-aortic lymphadenectomy up to the renal vessels [4]. This type of surgery carries considerable risks in most women with endometrial cancer at an apparent early presurgical stage [5]. Systematic lymphadenectomy is associated with surgical complications, especially in obese patients, who represent a significant percentage of women with endometrial cancer [6]. For this reason, the diagnostic capacity of intraoperative mapping of the sentinel lymph node has been studied and improved for more than 20 years [5,6]. At the same time, research has expanded into the field of diagnostic imaging to presurgically identify risk factors that may be associated with a more probable lymph node spread of endometrial cancer [7]. Obviously, it would be desirable to identify the risk factors associated with an advanced stage and/or a higher recurrence rate by analyzing the histology and grade of the tissue obtained from tumor biopsy at the time of the first diagnosis and, therefore, before planning the therapeutic interventions that can be proposed for each woman [3,8].

In the early 1980s, Bokhman [9] proposed dividing endometrial cancer into two categories: type I (low-grade endometrioid, typical of perimenopause and related to estrogenic hyperstimulation of the endometrial tissue, which is usually associated with a favorable prognosis) and type II (serous, typical of advanced postmenopause, unrelated to estrogenic stimulus and with a poorer prognosis). However, this classification did not allow for clear localization of grade III endometrioid carcinoma and clear cell carcinoma [9].

Less than ten years ago, a paper published by The Cancer Genome Atlas (TGCA) Research Network [10] revealed that endometrial cancer can be classified into four molecular categories according to prognostic significance and sensitivity to postsurgical adjuvant treatments. Unfortunately, this molecular classification cannot be easily used in clinical practice due to high costs and the need to use fresh or frozen tumor tissue [10]. For this reason, diagnostic algorithms combining immunohistochemical markers and molecular tests applicable to formalin-fixed, paraffin-embedded tumor tissue have been proposed and tested [11,12,13].

The progress achieved in the classification of endometrial cancer with diagnostic algorithms combining immunohistochemical markers and molecular tests has made it possible to better define, for prognostic purposes, the risk groups currently included in the latest European Society of Gynecological Oncology/European Society for Radiotherapy and Oncology/European Society of Pathology (ESGO/ESTRO/ESP) guidelines for the management of endometrial cancer [14]. To identify the prognostic categories equivalent to those obtainable with the molecular classification proposed by the TGCA, the diagnostic algorithm that is currently used, widely available and cost-effective is based on three immunohistochemical markers and a molecular test (p53, mismatch repair markers mutS homologue (MSH)-6 and postmeiotic segregation increased (PMS)-2 and a mutation analysis of the exonuclease domain of polymerase-ε (POLE)) [10,11,15]. Prospective clinical trials have all consistently confirmed the prognostic relevance of the diagnostic algorithm in high-grade endometrial cancers [16].

The consideration of other immunohistochemical markers, such as L1 Cell Adhesion Molecule (L1CAM), would still be valuable to better classify those cases of endometrial carcinoma that are still considered globally as low risk but have clinically different outcomes [17].

In this Special Issue, we invited experts in the field of endometrial cancer histopathology and immunohistochemistry as well as risk assessment and prognosis to contribute original articles or systematic reviews and meta-analyses reporting recent advances in the role of immunohistochemical markers in stratifying women with endometrial cancer into homogeneous prognostic groups who could benefit from specific individualized therapies.
